# Biopsy experience in a FAP endemic area

**DOI:** 10.1186/1750-1172-10-S1-I15

**Published:** 2015-11-02

**Authors:** Manuel Melo Pires

**Affiliations:** 1 Neuropathology Unit, Hospital Santo António / Centro Hospitalar do Porto, Porto, Portugal

## Background

Over a thousand nerve, skin and salivary gland biopsies were performed in FAP patients during the last forty years in our centre. We will try to demonstrate the different biopsy approaches over the years and confirm that biopsy of different types of tissue are useful not just in the diagnosis but also in the study of the pathogenesis of the disease.

## Methods

Retrospective analysis of our database since 1973 till 2014 showed 253 nerve biopsies, 441 skin and 738 salivary gland biopsies. Some of these were combined biopsies, nerve and skin or muscle nerve and skin. Except for the salivary gland biopsies they were all performed by one of our neuropathologists.

## Results

Until 1988, nerve and skin biopsies were commonly performed for diagnostic purposes, after that with genetic testing being available all tissue biopsies declined in number and were only needed in difficult diagnostic cases or when genetic testing was not available. Nerve biopsy became relevant again with the appearance of atypical clinical presentations or late onset or sporadic patients where the clinical diagnosis of FAP had not been considered. Nerve biopsy was also performed to establish the diagnosis of “de novo” amyloid neuropathy in recipients of domino liver transplants from FAP individuals, who started showing symptoms of neuropathy, and to rule out other causes of neuropathy.

Since 2005, salivary gland biopsy has become our method of choice to demonstrate amyloid and is as effective as nerve or skin biopsies.

## Conclusions

During the last forty years biopsies of different tissues were used to demonstrate amyloid. Nerve biopsy is still a useful tool not just for diagnostic purposes in problematic cases but also to understand the pathogenesis of the disease. Salivary gland biopsy is now our favoured method as it is a minimally invasive procedure and amyloid is demonstrated in very early stages of the disease. Biopsy could also be a tool to monitor treatment with the new drugs available.

